# American Ideals and Hospital Efficiency: Looking Forward

**Published:** 1915-10-09

**Authors:** 


### AMERICAN IDEALS AND HOSPITAL EFFICIENCY.

### LOOKING FORWARD.

In North America, hospital managers and members of hospital staffs are applying to the study of hospital efficiency that almost feverish originality, energy, and desire for constant improvement which the world has learned to regard as peculiarly characteristic of the American people. It may be, most probably it is, that the defects of their temperament and of their socio-political methods may also in practice modify the progress that results from this impatience of contentment with things as they are; and that voluntary hospital administration in the United States and in Canada may be thereby unfavourably influenced. But whether this is so or not, it cannot be denied that new ideas and improved methods are being sought for by hospital administrators across the Atlantic with more persistence than in the United Kingdom. On various occasions in past years we have commented on some of these tendencies and developments; it would be foolish indeed to ignore the lines along which modern expert thought is attempting to progress in so virile a people, especially as the American hospital system more closely approaches our own than perhaps that of any other nation does. Within the last two years, for instance, New York, Chicago, Boston, and Philadelphia have all produced reports and discussions directly concerned with problems of hospital efficiency and organisation. The Philadelphia County Medical Society has been particularly active in promoting co-operation between the various hospitals in that city, with a view to economy of time, labour, and money; the report of a committee appointed by this society stated that "the hospital should define clearly the rules and regulations governing the ordinary procedure for every department, so that even when there are frequent changes in the *personnel* of the medical staff, the nursing staff, or the administrative staff, the policy and practice of the hospital will be continuous."

### Scientific Management.

One of the latest communications on hospital improvement was read by Dr. R. L. Dickinson at a joint meeting of the Philadelphia and New York Obstetrical Societies in the former city; and is printed in the September number of the *American Journal of Obstetrics*. This gentleman favours the trial of a "science of management" which has been found to answer in factories and was suggested for adaptation to hospitals by Mr. Gilbreth at the 1914 meeting of the American Hospital Association. That speaker, himself an engineer, plainly intimated that his proposals might not be palatable to everyone. "You must submit," he said, "to having accurate measurements applied to your present methods and practices. You must recognise that for ages there has been a disinclination not only on the part of surgeons and doctors, but on the part of all those connected with a hospital, to allow their work to be inspected and the methods and results to be measured. . . . Another concession to be made is that no one is fitted to handle every kind of function of hospital management . . . also the necessity of specialising such functions as inspection and discipline."

### Staff Inspection.

Dr. Dickinson, after quoting these sentences from Mr. Gilbreth's paper, goes on to outline a plan for the scientific management of hospitals along these lines. He propounds a system of inspection and report which is certainly visionary at present, as far as Great Britain is concerned, but may be more within the range of practical politics a few years hence. Thus he would have an "efficiency rating" made for each individual worker in the hospital, analogous to that which in the United States appears to exist in the Civil Service. He puts forward a schedule for rating the usefulness of each member of the professional staff. This schedule is divided into seven sections, in each of which a large number of entries have to be made. Thus in Section A must be registered the total number of visits which the subject of the report should pay during a certain period; the number he did actually pay; the total number of hours given to the wards; the amount of vacation taken; the duration of any absences owing to illness and of any other absences, excused and unexcused; and the amount of committee service rendered. Section B endeavours under a great number of headings to estimate the quality of the work of each member of the staff, including his teaching of students as well as his clinical methods and results. Section C includes factors labelled "personality": such as integrity; disinterestedness; obedience to rules; team-play; kindness; cheerfulness; enthusiasm; and ability to inspire these. Then Section D involves "progressiveness; study of literature; travel to other clinics; membership and attendance at medical societies; originality; researches; publications." And so on through the other sections. Marks are to be assigned (by the inspector) on all these points, and by means of these marks a comparison of the values to the hospital of the different members of the staff is to be arrived at.

He desiderates two appointments (at a large hospital where teaching is undertaken), those of instructor and of inspector. Of the former we need only say that the outline of his duties put forward implies that he is to be essentially an educationist, concerned with improving medical educational methods and co-ordinating the work of all the subordinate teachers. Of the inspector, Dr. Dickinson would exact the following none too easy tasks: (1) To familiarise himself with the regulation of the hospital in general and with the written standard practice instructions of the professional staff in particular; (2) to inspect or to arrange for the inspection (at stated times) of all strictly medical or surgical laboratory work of the hospital; (3) to tabulate, summarise, and report upon conformity or nonconformity with the standards as in (1); (4) to prepare or cause to be prepared an efficiency record for each member of the professional staff and house staff and dispensary staff according to the schedule adopted by the professional staff and trustees; (5) to delegate to others certain parts of these inspections and reports if deemed wise, for example, (*a*) to consultant, on quality of work done by senior visiting staff; (*b*) to senior in service, on work done by his direct subordinates; (*c*) to dispensary committee, on work done by dispensary staff; (*d*) to superintendent of training school, on nursing in general.

It is easy enough to pick holes here and there in these far-reaching proposals; to point out, for instance, the loopholes for "eyewash" afforded in Section D of the schedule, and the glorious opportunities for self-advertisement which the astute would be sure to take advantage of. It must be admitted that the method would require men of very exceptional character, insight, energy, and honesty for the post of inspector, and that any failure therein would involve disaster. It is, again, quite clear that, since seniors report upon juniors, there is a good chance that tactfulness and opportunism—not to say gross flattery and obsequiousness—may obtain higher marks than do hard work, ability, and high professional efficiency. The latter complaint is, of course, not infrequently heard under the haphazard system, or want of system, which governs hospital staff appointments, in England. Those who have read this Journal during the last few years know that hospital inspection, apart from the purely voluntary and officially unrecognised efforts of the Editor, does not exist in this country as far as the voluntary hospitals are concerned. Few of those who know the facts would contend that inspection and control on some uniform plan would not militate in favour of progress, and on this ground, imperfect in its details as it certainly is, the broad principles of Dr. Dickinson's paper are well worthy of the attention of all who are interested in hospital management in this country.

